# Atypical negative pressure pulmonary edema with myocardial injury post-appendectomy: A case report

**DOI:** 10.1097/MD.0000000000046931

**Published:** 2026-01-02

**Authors:** Jingyuan Du, Ting He, Xuecong Wang, Jianbing Yang, ShiWu Wuer, Gebu Aer, Qi Wu, Tanqi Chen

**Affiliations:** a Cardiovascular Department, Bei Lun District People’s Hospital, Beilun District, Ningbo, Zhejiang, China; b Emergency Department, Beilun District Hospital of Traditional Chinese Medicine, Beilun District, Ningbo, Zhejiang, China; c Anesthesia Department, Beilun District Hospital of Traditional Chinese Medicine, Beilun District, Ningbo, Zhejiang, China.

**Keywords:** case report, catecholamine toxicity, inflammatory response, myocardial injury, negative pressure pulmonary edema

## Abstract

**Rationale:**

Negative pressure pulmonary edema (NPPE), an uncommon perioperative complication, primarily results from acute upper airway obstruction. While typically affecting young healthy patients with prominent respiratory manifestations, concurrent myocardial injury remains exceptionally rare. This case reports an atypical case of NPPE with biomarker-confirmed myocardial injury but absent respiratory symptoms.

**Patient concerns:**

A 26-year-old male developed a significant hemodynamic derangement following extubation after an appendectomy. Immediately post-extubation, he exhibited transient hypertension (150/100 mm Hg) and tachycardia (100 bpm), which progressed to sustained hypotension (86/47 mm Hg) within 50 minutes, persisting for 3 hours. Notably, this occurred in the complete absence of respiratory symptoms such as dyspnea or frothy sputum.

**Diagnoses:**

The diagnosis of NPPE was confirmed by chest computed tomography showing pulmonary edema. Concurrent myocardial injury was biomarker-confirmed (troponin I 2.49 ng/mL; creatine kinase–myocardial band 7.85 ng/mL), despite unremarkable echocardiography and electrocardiography.

**Interventions:**

Initial management for transient hypertension and tachycardia included intravenous urapidil and esmolol. For subsequent sustained hypotension, fluid resuscitation was administered. Following diagnosis, treatment involved diuretics and corticosteroids.

**Outcomes:**

The patient’s hemodynamic instability resolved, and his condition improved with the instituted therapy.

**Lessons:**

This case highlights that NPPE can present with occult myocardial injury without respiratory signs, necessitating serial cardiac biomarker monitoring in hemodynamically unstable patients. The underlying mechanism, while not directly proven here, may involve catecholamine surge, inflammatory cascades, and hypoperfusion.

## 1. Introduction

Negative pressure pulmonary edema (NPPE) is an uncommon non-cardiogenic complication following general anesthesia, with reported incidence of 0.05% to 0.1% in adults, although more recent data may suggest a higher rate.^[1,2]^ Typically affecting young healthy and physically fit individuals, it arises from acute upper airway obstruction such as laryngospasm, epiglottitis, or endotracheal tube obstruction and classically presents with dyspnea, wheezing, cyanosis, or pink frothy sputum within 6 hours postoperatively. The condition tends to resolve rapidly within 24 to 48 hours once the airway obstruction is relieved, and prognosis is generally favorable.^[2,3]^ Myocardial injury secondary to NPPE is exceptionally rare. We report a case of NPPE with biomarker-confirmed myocardial injury lacking typical respiratory symptoms.

## 2. Case report

### 2.1. Patient information

A 26-year-old previously healthy male presented with acute appendicitis complicated by localized peritonitis. No significant medical, family, or psychosocial history was documented.

### 2.2. Clinical findings

Preoperative examination revealed no abnormalities. The patient underwent emergent laparoscopic appendectomy under general anesthesia with tracheal intubation maintained for 72 minutes. The procedure itself was uncomplicated. The anesthesia regimen included: glycopyrronium 0.2 mg, sufentanil 30 μg, cisatracurium 14 mg, remifentanil 348 μg, propofol 535 mg, and sevoflurane. Intraoperative fluid administration consisted of 1000 mL crystalloid solution.

Immediately post-extubation, the patient exhibited a sudden, forceful inspiration accompanied by brief, mild inspiratory stridor, clinically consistent with transient laryngospasm or upper airway obstruction. This was immediately followed by hemodynamic fluctuations: blood pressure acutely elevated from 120/80 mm Hg to 150/100 mm Hg with a concomitant heart rate increase from 80 to 100 bpm. Intravenous urapidil (15 mg) and esmolol (20 mg) were administered. Within 50 minutes, hypotension developed (86/47 mm Hg) and persisted for 3 hours. The patient responded poorly to an initial rapid infusion of 500 mL of crystalloid solution. Consequently, an intravenous norepinephrine infusion was initiated at a maximum dose of 0.1 μg/kg/min to maintain a mean arterial pressure above 65 mm Hg. With continued fluid resuscitation (totaling 1500 mL of crystalloids) and vasopressor support, blood pressure gradually stabilized after approximately 3 hours. Vital signs during this hypotensive period were: temperature 37.0°C, heart rate 80 bpm, respiratory rate 13/min, peripheral oxygen saturation 99% on 3 L nasal cannula (fraction of inspired oxygen 33%). Notably, no ongoing respiratory distress, frothy sputum, or cough was observed. Serum lactate level during hypotension was not measured. Total urine output during the 3-hour hypotensive episode was approximately 80 mL.

### 2.3. Timeline

See Table 1.

**Table 1:** Clinical timeline, key findings, and interventions.

Time point	Event	Key findings	Interventions
Pre-op (05.15)	Normal chest X-ray (Fig. 1)	Unremarkable	None
Extubation (T = 0)	Forceful inspiration with stridor	BP ↑150/100 mm Hg, HR ↑100 bpm	Urapidil 15 mg, Esmolol 20 mg IV
T + 50 min	Hypotension onset	BP ↓86/47 mm Hg	Fluid resuscitation; Norepinephrine 10 µg IV
T + 3 h	Hypotension resolved	BP normalized	–
T + 6 h	Hospital transfer	CT: bilateral infiltrates (Fig. 2); elevated troponin I (2.49 ng/mL), CK-MB (7.85 ng/mL)	–
T + 17 h (05.16)	Treatment initiation	CT: edema improvement (Fig. 3)	Furosemide 20 mg IV →10 mg quaque 12 hora IV; methylprednisolone 40 mg IV quaque die
Day 4	Discharge	Biomarkers normalized	-

BP = blood pressure, CK-MB = creatine kinase-myocardial band, CT = computed tomography, HR = heart rate.

### 2.4. Diagnostic assessment

**Figure 1.**
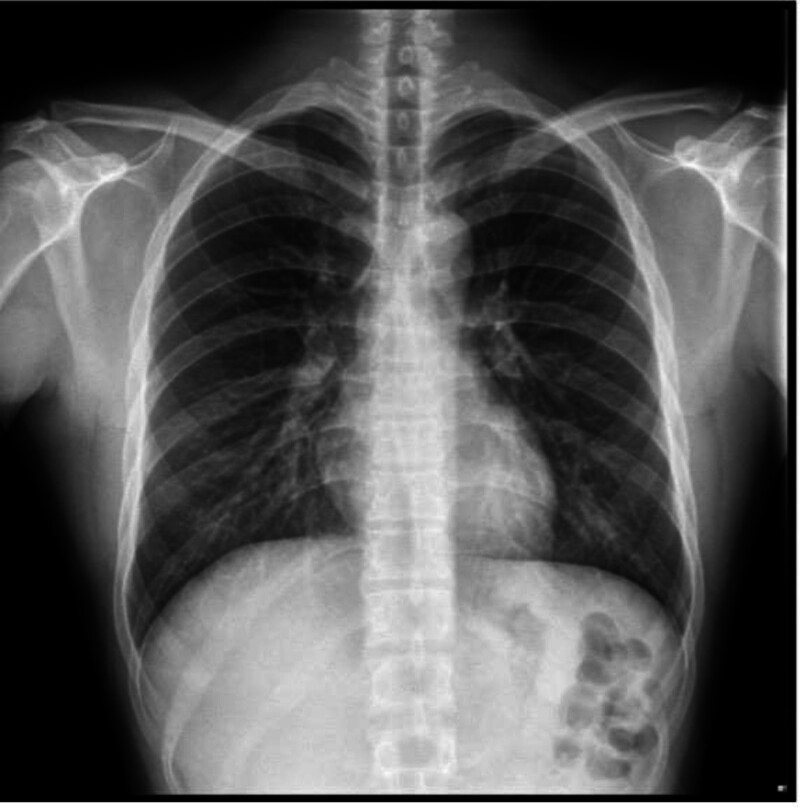
Preoperative chest radiograph (May 15, 2025): normal bilateral lung fields with preserved costophrenic angles and absence of consolidation.

**Figure 2.**
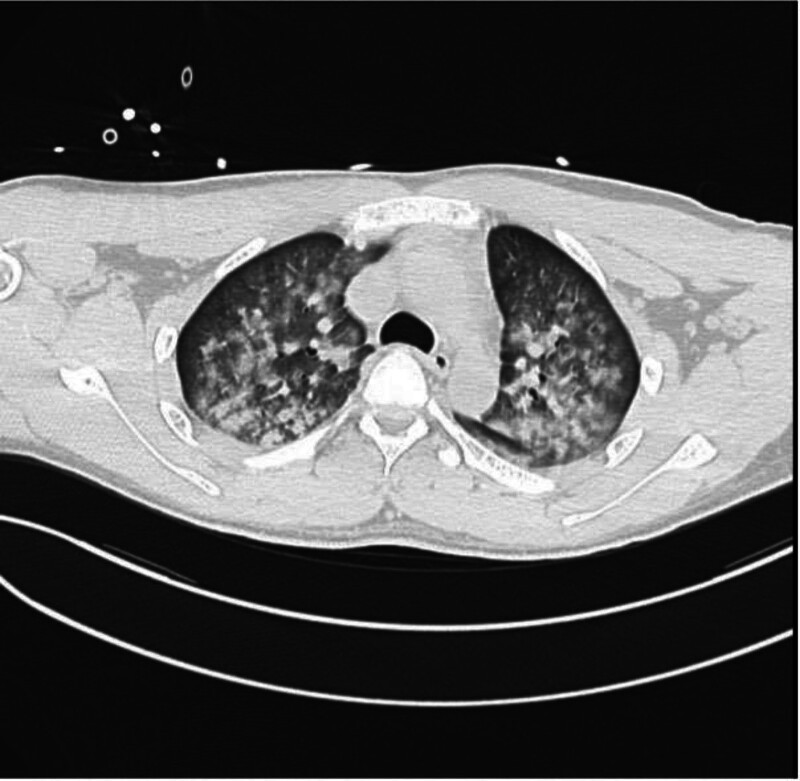
Non-contrast chest CT (May 15, 2025): extensive bilateral ground-glass opacities with peri-hilar and upper lobe predominance, 6 hours post-extubation. CT = computed tomography.

Contrasting imaging findings were critical:

• Preoperative chest radiograph: normal (Fig. 1).
• Postoperative computed tomography: bilateral extensive patchy infiltrates (Fig. 2).

Supporting laboratory data: creatine kinase-myocardial band 7.85 ng/mL, troponin I 2.49 ng/mL, D-dimer 120 μg/L, B-type natriuretic peptide 45 pg/mL. Noninvasive cardiac output monitoring (using the OMAY MED device) suggested myocardial involvement, yet echocardiography and electrocardiogram remained normal.

Differential diagnosis systematically excluded:

1. Cardiogenic edema (normal B-type natriuretic peptide, preserved ejection fraction, no cardiomegaly).
2. Neurogenic edema (absent neurological insults).
3. Allergic edema (no bronchospasm/rash, temporally discordant from drug exposure).

Final diagnosis: NPPE with associated myocardial injury. Prognosis was deemed favorable based on rapid biomarker normalization trend.

### 2.5. Therapeutic intervention

Management comprised:

• Furosemide: 20 mg IV bolus followed by 10 mg IV quaque 12 hora. The net fluid balance over the first 24 hours was approximately −400 mL.
• Methylprednisolone: 40 mg IV daily for 2 days.

The rationale for corticosteroid administration was the concern for a significant inflammatory component contributing to both the alveolar-capillary leak and the potential myocardial stunning in the context of profound hemodynamic stress, though we acknowledge this is not standard therapy.

### 2.6. Follow-up and outcomes

**Figure 3.**
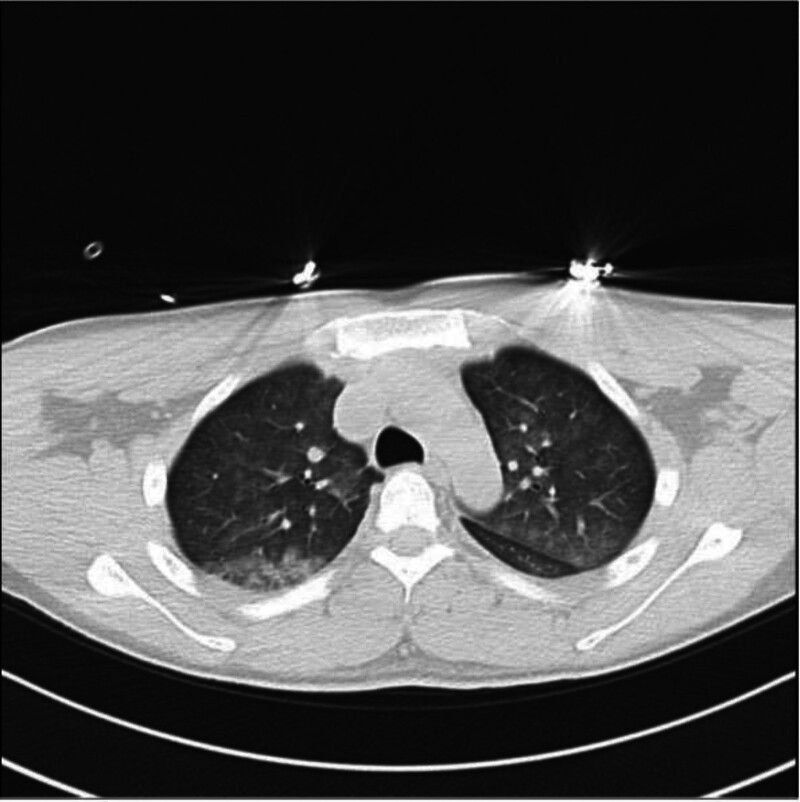
Follow-up chest CT (16 May, 2025): marked interval resolution of pulmonary edema 17 hours postoperatively. CT = computed tomography.

Clinical course demonstrated:

• Radiographic resolution at 48 hours (Fig. 3).
• Troponin normalization by postoperative day 4.
• Discharge on day 7 with stable hemodynamics.

The regimen was well-tolerated without adverse events.

## 3. Discussion

### 3.1. Pathophysiological framework

NPPE arises through dual interconnected pathways. The initial mechanism involves significant negative intrathoracic pressures (−50 to −140 cm H₂O) generated during airway obstruction, augmenting right ventricular preload and elevating pulmonary venous pressure. This hemodynamic shift reduces interstitial hydrostatic pressure, promoting fluid translocation, and hydrostatic pulmonary edema.^[4,5]^ Concurrently, mechanical stress from forceful inspiration damages alveolar-capillary membranes, increasing vascular permeability and facilitating protein-rich exudation into interstitial spaces – characteristic of permeability edema.^[6,7]^ Clinically, the diagnostic triad comprises: antecedent airway obstruction; self-limiting clinical course; and radiographic evidence of bilateral infiltrates or ground-glass opacities that typically resolve within 24 to 48 hours.^[8,9]^

### 3.2. Clinical management insights

In the present case, the diagnostic approach demonstrated notable strengths through rapid computed tomography imaging and serial cardiac biomarker assessment, enabling timely intervention. However, the absence of invasive hemodynamic monitoring (e.g., central venous pressure), lack of lactate measurement, and lack of histological confirmation via endomyocardial biopsy represent limitations in elucidating the precise myocardial injury mechanisms. The patient’s presentation diverged from classic NPPE descriptions by manifesting isolated hemodynamic instability without respiratory symptoms, aligning with emerging reports of “silent NPPE.”^[3]^ While myocardial biomarker elevation mirrored stress cardiomyopathy patterns,^[10,11]^ the absence of electrocardiographic abnormalities presents a distinctive clinical phenotype.

### 3.3. Mechanistic considerations for myocardial injury

We hypothesize that the significant myocardial injury in this young patient resulted from a synergistic insult. A catecholamine surge from sympathetic hyperactivity initiated myocardial damage through pathways of heightened oxygen demand, microvascular spasm, and direct cytotoxicity.^[12]^ This was compounded by a sustained period of systemic hypotension, leading to coronary hypoperfusion. Inflammatory cascades, triggered by alveolar-capillary barrier disruption and potentially by gut translocation due to peritonitis, may have further amplified neutrophil infiltration and secondary myocardial injury.^[13,14]^ The primary event was the upper airway obstruction, but the ensuing 3-hour hypotensive episode likely played a critical role in the extent of the myocardial damage.

### 3.4. Therapeutic implications and clinical lessons

Fundamental to NPPE management remains immediate relief of airway obstruction. Diuretic therapy requires judicious application due to risks of exacerbating hypovolemia, as highlighted by the patient’s muted response to fluid challenge. The use of corticosteroids for alveolar-capillary stabilization lacks robust clinical validation^[2,3,7]^ and was an individualized decision in this case based on the severity of presentation and concern for inflammatory-mediated injury; its benefit remains uncertain. This case underscores critical insights: NPPE may present exclusively through hemodynamic derangements; occult myocardial injury warrants systematic biomarker surveillance, especially after sustained hypotension; and the pathophysiological interplay between airway obstruction, catecholamine surge, and hypoperfusion merits heightened clinical vigilance.

## 4. Conclusion

NPPE constitutes an uncommon yet critical postoperative complication, typically precipitated by acute upper airway obstruction. We present a case suggestive of NPPE that was associated with subsequent myocardial injury in a previously healthy 26-year-old male following appendectomy. The prolonged hypotension observed is highly atypical for an uncomplicated NPPE course, suggesting the initial pulmonary event may have triggered a secondary hemodynamic cascade.

This case demonstrates that clinical features of NPPE may manifest atypically without classic respiratory symptoms, necessitating rigorous evaluation of post-extubation hemodynamic instability through serial biomarker assessment and dynamic imaging. The occurrence of occult myocardial injury in this context mandates systematic cardiac monitoring to prevent diagnostic oversight.

While myocardial involvement concurrent with NPPE remains rare, its potential pathogenesis may involve a complex interplay of catecholaminergic, inflammatory, and hemodynamic stressors. The exact mechanisms, however, require further elucidation. This complex presentation demands heightened clinical vigilance and warrants additional investigation into the underlying pathophysiology and potential long-term cardiovascular sequelae.

## Author contributions

**Conceptualization:** Jingyuan Du, Tanqi Chen, ShiWu Wuer.

**Data curation:** Jingyuan Du.

**Formal analysis:** Tanqi Chen, Ting He, Xuecong Wang.

**Investigation:** Jianbing Yang, Gebu Aer.

**Supervision:** Tanqi Chen.

**Writing – original draft:** Jingyuan Du, Qi Wu.

**Writing – review & editing:** Tanqi Chen.
